# Frailty syndrome and end-stage kidney disease outcomes at a Latin American dialysis center

**DOI:** 10.7705/biomedica.7057

**Published:** 2023-12-29

**Authors:** Luis David Moreno, Carlos Eduardo Ruiz, Juan Carlos Urrego, Miguel Oswaldo Cadena, Silvia José Maldonado, Daniel Andrés Niño, Andrea Maldonado

**Affiliations:** 1 Departamento de Medicina Interna, Hospital Universitario de Santander-Universidad Industrial de Santander, Bucaramanga, Colombia Universidad Industrial de Santander Departamento de Medicina Interna Hospital Universitario de Santander Universidad Industrial de Santander Bucaramanga Colombia

**Keywords:** Kidney diseases, frail, dialysis, renal replacement therapy, enfermedades renales, fragilidad, diálisis, terapia de reemplazo renal

## Abstract

**Introduction.:**

Frailty syndrome generates a high risk of adverse outcomes and mortality, and its prevalence is elevated in patients with end-stage kidney disease. Few studies have reported the prevalence and outcomes of frailty in populations from less developed countries.

**Objective.:**

To identify the clinical outcomes and factors associated with the frailty syndrome in patients with stage five chronic kidney disease who started renal replacement therapy - both hemodialysis and peritoneal dialysis- in a dialysis center in Bucaramanga, Colombia.

**Materials and methods.:**

This was a prospective study of patients with end-stage kidney disease who initiated dialysis at a center in Colombia and had a twelve-month follow-up.

**Results.:**

The overall frailty prevalence was 50.47% and two out of three patients older than 65 years had the syndrome. We found significantly higher followup mortality among patients with frailty: odds ratio of 2.95 (CI: 1.07- 8.13; p=0.036) in unadjusted analysis.

**Conclusions.:**

Literature shows that compared to developed nations, Latin American adults are facing a higher prevalence of chronic diseases, and frailty syndrome is increasing.

In this study, according to the FRAIL scale, having a frailty syndrome predicts a higher mortality; hypoalbuminemia and low creatinine levels at the beginning of dialysis could act as predictors of its diagnosis.

## Resumen

**Introducción.:**

El síndrome de fragilidad implica un alto riesgo de desenlaces adversos y mortalidad, y tiene una prevalencia elevada en pacientes con enfermedad renal en etapa terminal. Hay pocos estudios que investiguen la prevalencia y los desenlaces de este síndrome de fragilidad en las poblaciones de los países en desarrollo.

**Objetivo.:**

Identificar los desenlaces clínicos y los factores asociados al síndrome de fragilidad en los pacientes con enfermedad renal crónica en estadio cinco que inician terapia de reemplazo renal -con hemodiálisis o diálisis peritoneal- en un centro de diálisis de Bucaramanga, Colombia.

**Materiales y métodos.:**

Se trató de un estudio prospectivo de pacientes con enfermedad renal en etapa terminal que iniciaron diálisis en un centro de Colombia y a quienes se les hizo seguimiento durante doce meses.

**Resultados.:**

La prevalencia global del síndrome de fragilidad fue del 50,47 % y dos de cada tres pacientes mayores de 65 años lo presentaban. Se encontró una mortalidad significativamente mayor entre los pacientes con síndrome de fragilidad: razón de probabilidad de 2,95 (IC:1,07-8,13; p=0,036) en el análisis no ajustado.

**Conclusiones.:**

La literatura muestra que, en comparación con los países desarrollados, los adultos latinoamericanos presentan una mayor prevalencia de enfermedades crónicas y un aumento progresivo del síndrome de fragilidad. En este estudio, la fragilidad -según la escala FRAIL- predijo una mayor mortalidad. Además, la hipoalbuminemia y los niveles bajos de creatinina al inicio de la diálisis podrían actuar como elementos predictores de su diagnóstico.

The frailty syndrome is characterized by the difficulty of overcoming acute stressors due to the vulnerability generated by the decreased physiological reserve and organic dysfunction related to age and comorbidities. It was initially described and predominantly studied in older adults, in whom it is an independent risk predictor of comorbidity and mortality compared with pre-frail or vigorous patients ^1^^,^^2^. Frail patients have a higher risk of hospitalization for any cause (OR=1.9; CI 95%: 1.74-2.07) and death (OR=2.34; CI 95%: 1.77-3.09) ^3^^-^^5^ as well as other adverse outcomes, including falls, lack of mobility, physical limitations, respiratory impairment, and cognitive decline ^1^.

The prevalence of the frailty syndrome increases proportionally with age, ranging from 7 to 12% in over 65 years old and close to 25% in over 85 years of age ^6^. With aging population and improved medical care, there is an increasing prevalence of chronic diseases, such as patients with end-stage kidney disease who become dialysis dependent, in whom the prevalence of the frailty syndrome is nearly 70% ^7^ and represents a 2.6 times higher risk of mortality and a 1.4 times higher risk of hospitalization acting as an independent risk factor ^8^.

Few studies have investigated the prevalence of the frailty syndrome in populations from less developed countries. A recent systematic review and meta-analysis showed that Latin America is experiencing a rapid increase in the aging proportion, and its association with poor living standards increases the likelihood of having the frailty syndrome ^9^.

Even though end-stage kidney disease is a frequent public health issue, compared with studies of frailty in the general geriatric population, there are significantly fewer studies focusing on the frailty syndrome in chronic dialysis patients ^7^ and even more, very few studies have validated frailty scales in dialysis populations in developing countries ^10^^,^^11^.

Relevant studies have shown the need to determine the prevalence and impact of frailty in Latin American and Caribbean countries ^9^. Thus, this research aimed to analyze the relation of socio-demographic and clinical variables with the frailty syndrome diagnosis (using the FRAIL scale) in patients initiating hemodialysis or peritoneal dialysis due to end-stage kidney disease in a Latin American dialysis center. Also, we estimated the association of the frailty syndrome with the incidence of hospitalization and death during the first twelve months of follow-up.

## Materials and methods

### Design and population

We carried out an analytical, prospective, observational study, with nonprobabilistic sampling. We included all patients over 18 years with endstage kidney disease initiating hemodialysis or peritoneal dialysis within three months before the recruiting for the research at a dialysis center in Bucaramanga from June 2019 to July 2020 and followed up for twelve months.

This study complies with the Helsinki Declaration principles, local regulatory standards, and universal guidelines for good clinical practices. The protocol was evaluated and approved by the research ethics committee of the *Universidad Industrial de Santander.* This is a descriptive study without interventions, therefore considered risk-free.

### Definitions and variables

For the analysis, patients were divided into a frail and no frail group. The frail group included robust and prefrail according to the FRAIL scale. We used the FRAIL scale because of its simplicity, proven validity for frailty syndrome diagnosis, and validation for the Spanish language ^12^. This tool assessed five clinical variables, and each detected variable added one point to the scale. The subject is considered frail with three or more points, pre-frail with one or two points, and vigorous if the score is zero.

Baseline data were recorded for each patient at admission, including age, sex, occupation, body mass index, and medical history. Also, the values of the main laboratory tests at admission, like serum creatinine, blood urea nitrogen, intact parathyroid hormone, hemoglobin, corrected calcium, and serum albumin.

### Outcomes

Each participant had a one-year follow-up, from admission until death, medical discharge due to kidney function recuperation or administrative processes, or the end of the follow-up period. Vital status and date of death (when applicable) were obtained from the dialysis center. Hospitalizations during the year after enrollment were ascertained from the dialysis center and medical record review.

### Statistical analysis

The statistical analysis was performed using the Stata™, version 14.0. We expressed values as the mean plus or minus standard deviation (SD) or median and interquartile range (IQR) for continuous variables, and the percentage of the group for categorical variables. Categorical variables were compared using the x^2^ or Fisher’s test between frail and no-frail groups depending on the quantity of data. Continuous variables were compared using the t Student test for normal distributed data and Wilcoxon for no normal.

Frailty syndrome’s possible predictors were evaluated using logistic regression. The variables with statistical significance of p<0.05 upon univariate analysis were included in a multivariate analysis. Multivariate logistic regression results were presented as odd ratios (OR) with their 95% confidence intervals (CI). The KaplanMeier survival analysis and log-rank test were used to measure mortality in the frail and no-frail groups.

## Results

### Characteristics of the study population

**Figure 1:**
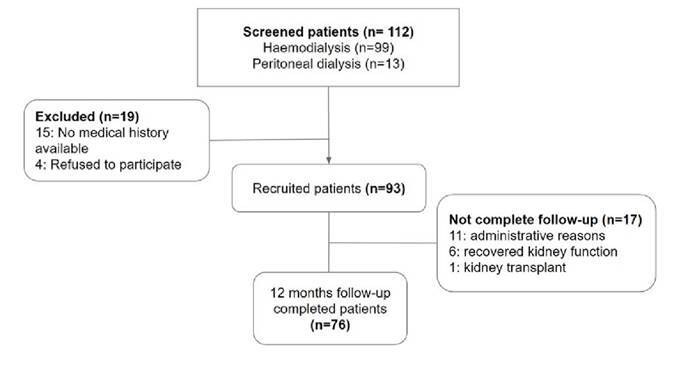
Study flow diagram showing 112 evaluated patients, 19 were excluded and 93 were included. Of the latter, 76 completed the follow-up.

A total of 93 patients starting dialysis during the follow-up period were included in the study (figure 1). The median age was 64 years (IQR: 53-69), and 59.14% were male. The mean Charlson comorbidity index was 6.17 ± 2.30: 81 patients (87.1%) had hypertension, 66 (70.97%) diabetes mellitus, and 26 (27.96%) chronic heart failure. Most patients (76.34%) started dialysis as an emergency, and just 11.83% initiated the peritoneal dialysis modality from the beginning. Only 76 patients completed the 12-month follow-up. The demographic and clinical characteristics of patients are summarized in table 1.

**Table 1:** Demographic, comorbidity, and laboratory characteristics according to frail status

Variable		Total (N=93)	Frail (n=47)	No frail (n=46)	p-value
Median age (years) (IQR)		64 (53-69)	67 (61-73)	59 (51-66)	<0.001
Gender					0.043 --
	Male [n (%)]	55 (59.14)	23 (48.94)	32 (69.57)	
	Female [n (%)]	38 (40.86)	24 (51.06)	14 (30.43)	--
Origin					0.144
	Rural [n (%)]	20 (21.51)	13 (27.66)	7 (15.22)	--
	Urban [n (%)]	73 (78.49)	34 (72.34)	39 (84.78)	--
Occupation and employment status					--
	Home [n (%)]	30 (32.26)	20 (42.55)	10 (21.74)	--
	Unemployed [n (%)]	12 (12.90)	8 (17.02)	4 (8.70)	--
	Merchant [n (%)]	7 (7.53)	0 (0)	7 (15.22)	--
	Farmer [n (%)]	6 (6.45)	4 (8.51)	2 (4.35)	--
	Other [n (%)]	38 (40.86)	15 (31.91)	23 (50)	--
	Labor active [n (%)]	51 (54.84)	19 (40.43)	32 (69.57)	0.005
Frail scale score					--
	0 [n (%)]	9 (9.68)	0	9 (19.57)	--
	1 [n (%)]	19 (20.43)	0	19 (41.3)	--
	2 [n (%)]	18 (19.3%)	0	18 (39.13)	--
	3 [n (%)]	14 (15.05)	14 (29.79)	0	--
	4 [n (%)]	23 (24.73)	23 (48.94)	0	--
	5 [n (%)]	10 (10.75)	10 (21.28)	0	--
	Charlson comorbidity index [Mean ± SD]	6.17 ± 2.30	7.06 ± 2.11	5.26 ± 2.14	<0.001
Arterial hypertension [n (%)]		81 (87.1)	40 (85.11)	41 (89.13)	0.563
Diabetes [n (%)]		66 (70.97)	37 (78.72)	29 (63.04)	0.096
Ischemic heart disease [n (%)]		17 (18.28)	13 (27.66)	4 (8.70)	0.03
Chronic heart failure [n (%)]		26 (27.96)	21 (44.68)	5 (10.87)	<0.001
Peripheral arterial disease [n (%)]		17 (18.28)	11 (23.4)	6 (13.04)	0.196
Stroke [n (%)]		8 (8.60)	5 (10.64)	3 (6.52)	0.479
Chronic obstructive pulmonary disease [n (%)]		5 (5.38)	3 (6.38)	2 (4.35)	0.664
Blood urea nitrogen [Mean ± SD]		54.55 ± 17.9	55.3 ± 20.12	53.8 ± 15.5	0.688
Creatinine Median (IQR)		5.65 (4.32-7.09)	5 (3.76-6.18)	6.95 (5.16-7.9)	<0.001
Hemoglobin [Mean ± SD]		9.59 ± 1.46	9.66 ± 1.47	9.52 ± 1,46	0.6663
Corrected serum calcium [Mean ± SD]		8.74 (8.1-9.14)	8.83 (8.57-9.17)	8.56 (7.89-9.02)	0.019
Serum albumin [Mean ± SD]		3.44 ± 0.59	3.29 ± 0.61	3.59 ± 0.54	0.015
Serum parathyroid hormone [Mean ± SD]		246.4 (170.7-387.3)	223.4 (164.4-316.3)	295.85 (207.9-493.3)	0.012

IQR: Interquartile range; SD: Standard deviation

### Prevalence, characteristics, and factors related to frailty

The overall prevalence of the frailty syndrome at the baseline was 50.54% (n=47). Nine patients (9.68%) were vigorous, and 37 (39.78%) were prefrail. The frailty syndrome patient’s median age was 67 (IQR: 61-73) years, and 48.94% were men. Women were more likely to be frail than men (63.16 % of females vs. 41.82 % of males; p=0.043). Frailty prevalence was 66.67 % in participants above 65 years old and 35.42 % in the group under 65 years old. Frail patients tended to be older (median=67; IQR: 61-73; p=0.0003) than non-frail patients (median=59; IQR: 51-66) and had a higher Charlson comorbidity index score (mean=7.06 ± 2.11; p=0.0001) compared to non-frail participants (mean=5.26 ± 2.14).

A higher proportion of non-frail patients worked actively (69.57%) compared to frailty ones (40.43%), while a higher proportion of frail patients had ischemic cardiomyopathy and chronic heart failure. Also, the frail group had lower values of serum albumin and serum creatinine, with statistically significant differences (table 1).

### Predictors of frailty

In the multivariate analysis, we found that ischemic heart disease was statistically associated with the frailty syndrome (OR=3.86; 95% CI: 1.0913.65; p=0.036). In the clinical laboratories analyzed, serum creatinine lower or equal to five was related to frailty (OR=3.26; CI: 1.09-9.73; p=0.035), as well as albuminemia levels lower or equal to 3.4 (OR=2.93; CI: 1.05-8.19; p=0.04) (table 2).

**Table 2:** Bivariate and multivariate analysis of factors related to frailty syndrome.

Variable	Bivariate		Multivariate		
Demographics and comorbidities	OR (95% CI)		p-value	OR (95% CI)	p-value
Female gender	2.39)	(1.02-5.58	0.045	--	--
Age (≥65 years)	3.65	(1.55-8.59)	0.003	--	--
Ischemic cardiomyopathy	4.01	(1.199-13.44)	0.024 3.86	(1.09-13.65)	0.036
Chronic heart failure	6.62	(2.22-19.74)	<0.001	--	
Charlson index (≥7 points)	5.98	(2.44-14.66)	<0.001	--	
Creatinine (≥5)	3.62	(1.49-8.78)	0.005 3.26	(1.09- 9.73)	0.035
Serum albumin (≥3.4)	4.03	(1.698-9.58)	0.002 2.93	(1.05-8.19)	0.04
Labor active	0.297	(0.13-0.69)	0.005	--	--

### Frailty syndrome patients’ outcomes

We followed up 76 patients during 12 months. Eleven patients (14.47%) were lost in the follow-up for administrative reasons, six patients (6.45%) recovered kidney function before the year of follow-up time, and one patient had a kidney transplant (figure 1).

**Figure 2:**
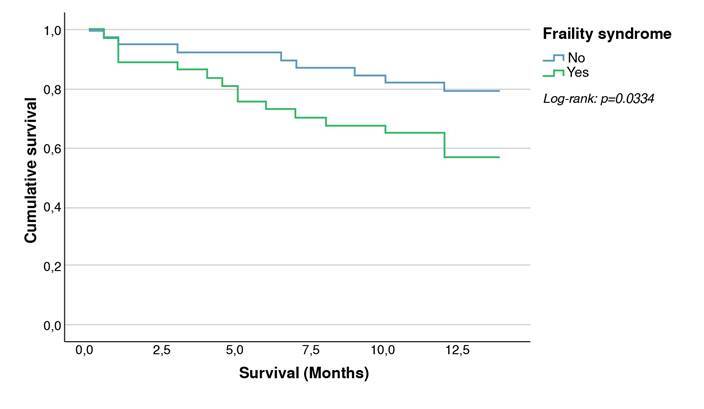
Kaplan-Meier survival by frail and no frail. (Log Rank: p-value =0.0334). One year follow-up survival among patients with frailty syndrome (56.76%) is lower than that of patients without frailty syndrome (79.49%).

Twenty-four participants (31.58%) died with a follow-up mean time to death of 5.88 ± 4.05 months. We found a significantly higher follow-up mortality among patients with frailty syndrome (43.24%) than non-frail (20.51%; p=0.033) with an OR=2.95 (95% CI: 1.07-8.13; p=0.036) in unadjusted analysis (figure 2).

In the year after enrollment, 36 (47.37%) participants had one or more hospitalizations. The median number of hospitalizations was two (IQR: 1-2), and the maximum was five. The median time of the first hospitalization occurrence was two months (IQR: 0.75-7). The proportion of patients with one or more hospitalizations was 51.35% for the frail group and 43.59% (p=0.498) for non-frail.

The most common cause of hospitalization was infections with 31 events (n=22; 28.95%), followed by cardiovascular causes with 16 events (n=13; 17.11%), decompensation of chronic diseases with 8 events (n=8; 10.53%), and related dialysis complications with 5 events (n=5; 6.58%) (table 3).

**Table 3:** Complications in the 12 month-follow up after starting dialysis

Variable	Frail patients (n=37) n (%)	No frail (n=39) n (%)	p-value
Deceased	16 (43.24)	8 (20.51)	0.033
Any hospitalization cause	19 (51.35)	17 (43.59)	0.498
Cardiovascular hospitalization	7 (18.92)	6 (15.38)	0.683
Heart failure	5 (13.51)	3 (7.69)	0.475
Acute myocardial infarction	1 (2.7)	2 (5.13)	1
Stroke	1 (2.7)	0	0.487
Hospitalization due to infections	13 (35.14)	9 (23.08)	0.247
Haemodialysis catheter-related infections	3 (8.11)	2 (5.26)	0.674
Peritoneal dialysis-related peritonitis	2 (5.41)	3 (7.69)	1
Skin and soft-tissue infections	2 (5.41)	3 (7.69)	1
Diabetic foot disease	3 (8.11)	0	0.111
COVID-19	2 (5.41)	6 (15.38)	0.263
Other causes of hospitalization	7 (18.92)	9 (23.08)	0.657
Number of hospitalizations	2 (1-2)	2 (1-2)	0.6708
Median (IQR=)			

## Discussion

Aging is becoming a very important issue in medical and political decisions worldwide. Nevertheless, populations of low- and particularly middle-income countries, including Latin Americans, are aging more rapidly than any country in the past. Twothirds of the world’s older people live in low- and middleincome countries, rising to 80% by 2050. Moreover, compared to developed nations, Latin American adults are facing a higher number of chronic diseases, including end-stage kidney disease ^9^^-^^10^. The present study is one of the few investigating the prevalence and impact of frailty in patients starting dialysis in a Latin American country.

The frailty syndrome prevalence in our study was 50.54% using the FRAIL scale, higher than the prevalence found by others, such as Jegatheswaran *et al.* (15%), using the same scale ^13^. This difference could be related to the fact that they excluded patients with some degree of physical, visual, or hearing disability, but principally, because most patients in our study started dialysis on an emergency basis, so they did not have access to a planned therapy initiation. Only 23% of our studied population started a dialysis plan in contrast to 76% who did it as an emergency measure. It is well known that dialysis initiation in the emergency room is associated with worse clinical outcomes, such as substantially higher mortality on admission and lower survival in the follow-up ^14^.

The median age in our study was 64 years, and the frail population was significantly older (p=0.0003), data similar to other regions’ reports ^15^. We found that the proportion of women with frailty syndrome was higher than in men (63.16% vs 41.82%). This finding is consistent with the reports of Johansen *et al.*^7^ and Baback *et al*. ^16^, concluding that women tend to be more fragile in all age groups. This gender difference has been suggested in many studies associating higher female prevalence of non-lethal diseases or due to differential biological factors, such as inflammatory cytokines, sarcopenia, and cognitive impairment.

In addition, we identified that frail patients abandoned their work activities more frequently than non-frail individuals. It could be related to higher cognitive impairment in the first group, as also described by McAdams- DeMarco *et al.*^1^.

The mean Charlson comorbidity index was 6.17 ± 2.30. The calculation for each group showed it was higher for the frail population (7.06 ± 2.11) than in non-frail patients (5.26 ± 2.14). These observations agree with those of García *et al*. They described a higher Charlson comorbidity index in frail versus non-frail patients (7.9 vs 4.7; p<0.001) ^17^. It contrasts with Rubio *et al*. and Huidobro *et al*. studies reporting no difference in the Charlson comorbidity index between the frail and non-frail group independent of the dialysis modality ^18^^,^^19^.

In the analysis of comorbidities, ischemic heart disease and heart failure were the most associated with the frailty syndrome. Bao *et al.*^20^ also found statistical significance with these two diseases and additionally in diabetes mellitus, peripheral arterial disease, and chronic obstructive pulmonary disease. Meanwhile, Jegatheswaran *et al.*^7^ and Johansen *et al*. ^13^ found that diabetes mellitus was the most prevalent condition in frail people (p<001).

Numerous studies have found that hypoalbuminemia was correlated with frailty regardless of the scale used for its diagnosis ^7^^,^^16^^,^^17^. In our study, lower levels of albuminemia were more common in frail patients (3.29 ± 0.61) as compared to non-frail patients (3.59 ± 0.54) with a statistically significant difference (p=0.0150). Similarly, lower levels of creatinine were found in patients with frailty. This fact has been explained by the lower production of creatinine related to sarcopenia despite the decrease in the glomerular filtration rate of kidney disease.

Large-scale prospective frailty studies have found a high risk of death in frail populations (1.71-2.24) ^21^^,^^22^. The frailty syndrome has been associated with a 2.5-fold increased risk of death and progression of chronic kidney disease, being these two conditions independent mortality risk factors ^16^^,^^23^. Johanssen *et al.*^7^ reported a 2.24 higher risk of death in dialysis patients, like McAdams report (2.6 times; 95% CI: 1.04- 6.49; p=0.04) (1) and Lee (2.37 times; 95% CI: 1.11- 5.02) ^24^. In our study, we found a higher mortality proportion in frailty people compared with non-frail patients during the 12-month follow-up (43.24% vs 20.51%; p=0.033) with an OR=2.95, 95% CI: 1.07-8.1, and p=0.036 in the unadjusted analysis. These data are similar to literature reports and higher than the unadjusted analysis realized by Bao *et al*. (HR=1.79; 95% CI: 1.44- 2.24; p<0.001) ^20^.

Like mortality, the frailty syndrome has been associated with a higher risk of hospitalization in the general population and end-stage kidney disease patients (^16^^,^^21^^,^^22^. McAdams *et al.* found a 1.43 (95% CI: 1.00-2.03; p=0.049) higher risk of hospitalization in frail patients using the FRAIL criteria ^1^. But intermediate frailty status was not associated with increased risk (RR=0.76; 95% CI: 0.49-1.16; p=0.21). Bao *et al.* found a 1.44 higher risk of first hospitalization in frailty syndrome patients (95% CI: 1.26-1.66; p<0.001) ^20^. In our study, 51.35% of frail and 43.59% of nonfrail patients had one or more hospitalizations during the follow-up period, with a non-significant statistical difference (p=0.498).

However, it is striking the high proportion of patients without frailty syndrome that have complications requiring hospitalization. Non-frail patients had a higher rate of hospitalization in the “other causes” category, which included complications related to dialysis modalities, like catheters, infections, and underlying disease decompensation. We consider that frail patients did not present a higher proportion of hospitalizations, not because they do not have a higher risk of complications, but because of the differential exposure to risks with respect to the non-frail group. In this regard, the nonfrail patients were mostly young people, actively working and in a low-resource setting, which could be a risk factor for poor adherence to treatment and exposure to complication triggers.

In conclusion, the frailty syndrome in dialysis-dependent patients predisposes to adverse outcomes such as hospitalization, reduced quality of life, and death. This study classified frailty degree using the FRAIL scale at a Latin American dialysis center and found a prevalence like those reported in other studies that used the FRAIL criteria for its diagnosis. Mortality in the first year of dialysis was higher for frail patients. New research is needed to validate the hypothesis of increased complications and hospitalizations according to exposure risk factors in dialysis patients, such as work status.

Despite the absence of a consensus about the best way to measure frailty, outcome identification, and associated factors will help to improve prognosis, timely interventions, and provider-to-patient communication ^25^. Also, according to the literature, Latin American countries will need to adapt their institutions and public policies to the new challenges that arise from a less healthy older population because some of those factors are potentially amenable to influence from public health and social care interventions ^9^^,^^10^.
